# Histone Deacetylases in the Pathogenesis of Diabetic Cardiomyopathy

**DOI:** 10.3389/fendo.2021.679655

**Published:** 2021-07-22

**Authors:** Xiangyu Ke, Zhirui Lin, Zebing Ye, Meifang Leng, Bo Chen, Chunjie Jiang, Xiaoyun Jiang, Guowei Li

**Affiliations:** ^1^ Centre of Clinical Epidemiology and Methodology, Guangdong Second Provincial General Hospital, Guangzhou, China; ^2^ Department of Cardiology, Guangdong Second Provincial General Hospital, Guangzhou, China; ^3^ Department of Endocrinology, Guangdong Second Provincial General Hospital, Guangzhou, China; ^4^ Department of Nutrition and Food Hygiene, School of Public Health, Tongji Medical College, Huazhong University of Science and Technology, Wuhan, China; ^5^ Department of Pulmonary and Critical Care Medicine, Guangdong Second Provincial General Hospital, Guangzhou, China; ^6^ Department of Health Research Methods, Evidence, and Impact, McMaster University, Hamilton, ON, Canada

**Keywords:** diabetic cardiomyopathy, histone deacetylases, cardiac fibrosis, cardiac hypertrophy, oxidative stress, inflammation

## Abstract

The global burden of diabetes mellitus and its complications are currently increasing. Diabetic cardiomyopathy (DCM) is the main cause of diabetes mellitus associated morbidity and mortality; therefore, a comprehensive understanding of DCM development is required for more effective treatment. A disorder of epigenetic posttranscriptional modification of histones in chromatin has been reported to be associated with the pathology of DCM. Recent studies have implicated that histone deacetylases could regulate cardiovascular and metabolic diseases in cellular processes including cardiac fibrosis, hypertrophy, oxidative stress and inflammation. Therefore in this review, we summarized the roles of histone deacetylases in the pathogenesis of DCM, aiming to provide insights into exploring potential preventative and therapeutic strategies of DCM.

## Introduction

Diabetes mellitus is a metabolic disease characterized by hyperglycemia. With the improvement of living standard, the incidence of diabetes mellitus continues to rise across the world (1). Diabetes mellitus causes long-term damage to multiple organs, ultimately leading to severe complications. Moreover, diabetes mellitus affects the heart through various mechanisms including metabolic disorder, microvascular injury, cardiac autonomic dysfunction, and a maladaptive immune response (2). Diabetic cardiomyopathy (DCM) is a common and severe complication of diabetes mellitus and increases the risk of heart failure with heavy social and economic burden worldwide (3, 4). DCM is a clinical problem which is present in both type 1 and 2 diabetes (5). People with diabetes (30%) always have more than twice the risk of developing heart failure as compared to people without diabetes (23%). Emerging evidence disclosed that 19-26% of diabetic patients were prone to develop heart failure (6, 7). DCM is characterized by left ventricular hypertrophy, cardiac fibrosis, chronic inflammation in the absence of hypertension, coronary, and other heart diseases in diabetic patients (4, 8, 9), ranging from left ventricular fibrosis and diastolic cardiac dysfunction in the early stage, to severe diastolic heart failure with normal ejection fraction (HFpEF, EF<40%) and ultimately to systolic dysfunction accompanied by heart failure with reduced ejection fraction (HFrEF, EF<50%) (10, 11). Importantly, DCM also increases the risks for the development of extensive focal myocardial necrosis, shock and sudden death. Recent studies have highlighted that a complex interplay between genes and environment may significantly contribute to pathogenesis of microvascular complications associated with diabetes (12). Several potential mechanisms that may contribute to the pathogenesis of the DCM have been proposed, including cardiac structural abnormalities, metabolic disturbances, mitochondrial damage, oxidative stress, autophagy/mitophagy defect, apoptosis, systemic inflammation, epigenetic modification, dampened coronary flow reserve, coronary microvascular disease (microangiopathy), and endothelial impairment (5, 13–17).

A concerted definition of epigenetic trait, “stably heritable phenotype resulting from changes in a chromosome without alterations in the DNA sequence”, was reached at a Cold Spring Harbor meeting (18–20). There are three major epigenetic modifications: histone modifications, DNA methylation and microRNAs (21). Histone acetylation which is the best-characterized histone modifications and is controlled by histone acetyltransferases (HATs) and histone deacetylases (HDACs). Histone deacetylases (HDACs) are a family of enzymes that are important regulators of epigenetic gene modification (22). Accumulating evidence has implicated that HDACs are associated with many cardiovascular diseases (CVDs) (19) including hypertension (23, 24), DCM (25, 26), myocardial infarction (27–29) and atrial fibrillation (30). HDACs have been implicated in numerous cellular processes relevant to DCM, which include cardiac fibrosis, hypertrophy, inflammation and oxidative stress (31). However, only quite a few publications have reported the potential impact of HDACs on DCM. In this review, we comprehensively reviewed the roles of HDACs in cellular processes relevant to DCM, aiming to discuss the implication of HDACs in the pathogenesis of DCM and provide insights into exploring potential preventative and therapeutic strategies of DCM.

## Epigenetics in DCM

Epigenetic mechanisms such as histone modification, DNA methylation and microRNA changes may play an important role in the development of DCM (32–34). Previous studies suggested that factors possibly implicated in the pathogenesis of DCM include cardiac fibrosis, cardiac hypertrophy, oxidative stress, and inflammation, which may contribute to alterations in the pathogenic gene expression by epigenetic mechanisms to initiate the pathogenic changes in the target cells and organs (35).

Reversible modifications of histones indicate covalent posttranslational modification of histone proteins, including methylation, acetylation, phosphorylation, ubiquitination and sumoylation (21). Histone modification, especially histone acetylation, is a major epigenetic mechanism regulating gene expression. Histone acetyltransferases (HATs) and histone deacetylases (HDACs), which catalyze histone acetylation resulting in gene transcriptional activation and remove acetyl groups resulting in gene silencing respectively, are the major players in maintaining the equilibrium of histone acetylation (8, 36).

To date, there have been 18 HDACs reported that can be divided into four categories based on their sequence identity and catalytic activity (37). Descriptions of the classification, cellular localization, substrates and main biological functions of HDACs are shown in **Table 1**. Brief summary of the four class HDACs are also mentioned below.

- *Class I* HDACs contain HDAC1, HDAC2, HDAC3 and HDAC8.
- *Class II* HDACs are subdivided into Class IIa (HDAC 4, 5, 7 and 9) and Class IIb (HDAC 6 and 10) (81, 82).
- *Class III* HDACs are called sirtuins sharing sequence homology with the yeast Sirt 2 protein, which contains seven sirtuin members, namely, SIRT1-SIRT7 (83). This highly conserved class of proteins thereby controls a range of different biological processes (84, 85).
- *Class IV* HDACs include a solitary member HDAC 11, which shares sequence homology with the yeast RPD 3 and HAD 1 proteins.

**Table 1:** The HDAC family: classification, cellular localization and substrates.

Class	Member	Localization	Substrates	Biological functions	Reference
I	HDAC 1	Nucleus	Histones, p53, MEF2, NF-κB, ATM,AR, BRCA1, pRb,	Cell proliferation, cell survival	(38–40)
	HDAC 2	Nucleus	Histones, HOP, NF-κB, GATA2, BRCA1, pRb, MECP IRS-1	Cell proliferation, insulin resistance	(38, 41)
	HDAC 3	Nucleus	Histones, HDAC4, 5, 7–9, SHP, GATA-2, NF-kB, pRb	Cell proliferation, cell survival	(38, 42)
	HDAC 8	Nucleus	Hsp70、PKM2	Cell proliferation	(38, 43)
IIa	HDAC 4	Nucleocytoplasmic traffic	Histones, MEF2,PGC-1α,Hsp70, p53,p21,GATA, FOXO, HIF-1α	Regulation of skeletogenesis and gluconeogenesis	(44–46)
	HDAC 5	Nucleocytoplasmic traffic	Histones, MEF2, HDAC3, YY1,NRF 2	Cardiovascular growth and function; cardiac myocytes and endothelial cell function; gluconeogenesis	(38, 47–49)
	HDAC 7	Nucleocytoplasmic traffic	Histones, MEF2, HDAC3, HIF-1α	Regulation of gluconeogenesis	(38, 50, 51)
	HDAC 9	Nucleus	Histones, MEF2, HDAC3	Cardiovascular growth and function	(49, 52)
IIb	HDAC 6	Cytoplasm	Tubulin, HSP90, HDAC11	Homologous recombination	(38, 53)
	HDAC 10	Nucleocytoplasmic traffic	LcoR, PP1	Cell survival, DNA damage repair,	(54, 55)
III	SIRT1	Nucleus, cytoplasm	Histones, p53, p300, MMP9, NF-κB, FOXO3A, AMPK, SERCA2a, PGC-1α,FGF21	Cell proliferation, cell survival, apoptosis, senescence, DNA repair, cell metabolism, calorie restriction	(56, 57)
	SIRT2	Cytoplasm	Histone H3, α-tubulin, FOXO1, FOXO 3a, NF-κB, AKT/GSK 3β, H4K16	Mitosis regulation, genome integrity, cell differentiation, cell homeostasis, aging, infection, inflammation, autophagy	(58–60)
	SIRT3	Mitochondria	Histones, Ku70,IDH2,HMGCS2, GDH, AceCS, SdhA, SOD2, LCAD	Glucose and fatty acid metabolism, apoptosis, tricarboxylic acid (TCA) cycle, oxidative stress	(61–63)
	SIRT4	Mitochondria	GDH	Cell metabolism, DNA damage responses	(64–66)
	SIRT5	Mitochondria	Cytochrome c, CPS1, NRF2, FOXO3A	Energy metabolism	(67–70)
	SIRT6	Nucleus	Histone H3, TNF-α, PKM2, PGC-1a, FOXO3	DNA damage response, inflammation, metabolism, genome maintenance	(71–74)
	SIRT7	Nucleolus	P53, histone H3	rDNA transcription, lipid metabolism, DNA damage repair	(75–77)
IV	HDAC11	Nucleus	Histones, HDAC6, Cdt1	Immunomodulators–DNA replication	(78–80)

MEF 2, myocyte enhancer factor 2; NF-κB, Nuclear transcription factor-kappa B; AR, Androgen receptor; ATM, Ataxia-telangiectasia-mutated; BRCA1, Breast cancer; pRb, Retinoblastoma protein; HOP, Homeodomain only protein; GATA, GATA binding protein; MECP, Methyl-CpG-binding domain protein; IRS, Insulin receptor substrate; SHP, Src homology region 2-domaincontaining phosphatase; Hsp70, Heat shock protein 70; PGC, Peroxisome proliferator-activated receptor gamma coactivator; HIF, Hypoxia-inducible factor; YY1, Yin Yang 1; NRF 2, nuclear factor erythroid 2-related factor 2; LcoR, ligand-dependent receptor co-repressor; PP1, Protein phosphatase 1; FOXO3A, Fork head box O3A; FOXO 1, Fork head box 1; HMGCS2, Mitochondrial 3-hydroxy-3-methylglutaryl-CoA synthase; GDH, glucose dehydrogenase; AceCS, Acetylcoenzyme A synthetase; SdhA, Succinate dehydrogenase complex subunit A; SOD, Superoxide dismutase; LCAD, Long chain acyl coenzyme A dehydrogenase; GPS1, Gravity Persistent Signal 1; TNF-α, tumor necrosis factor-α; PKM2, pyruvate kinase M2;.

## HDACs, Cardiac Fibrosis and DCM

Cardiac fibrosis is a hallmark of DCM and is caused by excessive matrix (ECM) proteins accumulation including collagen I and collagen II. Fibrosis increases the passive stiffness of the myocardium and impairs relaxation and diastolic dysfunction (86). Elevated perivascular and intermyofibrillar fibrosis has been observed in human myocardial samples in the absence of coronary heart disease and hypertension (87, 88). This further illustrates the presence of myocardial fibrosis in diabetic cardiomyopathy.

HDACs are emerging as crucial regulators of cardiac fibrosis, although the cellular mechanisms by which HDACs regulate cardiac fibrosis have not been fully understood (89). Current studies provide insufficient evidence for the role of HDACs in DCM; however sizable explorations have reported that HDACs were dysregulated in cardiac fibrosis (29, 90). For instance, SIRT6 knockout mice presented cardiac fibrosis and dysfunction with cardiomyocyte hypertrophy and increased apoptosis (90). Recently, SIRT1, as a protein regulator, has attracted widespread attention because of its salutary effect in DCM (56). One study found that SIRT1 alleviated cardiac fibrosis in the development of DCM. Specifically, bakuchiol (BAK) alleviated cardiac fibrosis in DCM *via* SIRT1-induced inhibition of ROS generation. Moreover, the TGF-β1/Smad3 signaling pathway played a key role in mediating ROS generation to pathologic fibrosis (91). Likewise, recent researches indicated that Class IIa HDACs could also own profibrotic functions (92–95). Zhang et al. found that the overexpression of activated HDAC4 exacerbated cardiac dysfunction and interstitial fibrosis in the model of myocardial infarction (29). Another study showed that HDAC 4 knockdown blocked cardiac fibrosis by inhibiting the expression of α-SMA and the phosphorylation of ERK (96). It is probably that HDAC4 is adverse to the development of DCM, but whether it will exacerbate cardiac fibrosis in DCM needs to be further explored.

HDACs inhibitors have been reported to be efficacious in rodent models of heart failure. By blocking pathological cardiac hypertrophy and fibrosis, HDACs inhibitors can improve cardiac function (97, 98). For example, MPT0E014 (a Class I/IIb HDAC inhibitor) attenuated cardiac fibrosis with heart failure induced by isoproterenol administration in rats (99). It was associated with downregulation of Ang II type I receptor (AT1R) and transforming growth factor-β (TGF-β) (99). HDACs inhibitors also have been reported to show protective effects on the diabetic heart (25, 26). Xu et al. found that selective inhibitor RGFP966 of HDAC3 ameliorated diabetes-induced fibrosis and deterred the development of DCM by obstructing the enhanced phosphorylated ERK1/2, and upregulating dual specificity phosphatase 5 (DUSP5) expression through increased acetylated histone H3 on the primer region of DUSP5 gene (26). Likewise, Chen et al. reported that the protective effects of HDACs inhibitor (sodium butyrate) in the diabetic myocardium were closely related to mitigating apoptosis, stimulating angiogenesis and increased SOD1 (25). Thus, their findings indicated that HDACs inhibitor had the potential to alleviate cardiac fibrosis and prevent the development of DCM (25).

## HDACs, Cardiac Hypertrophy and DCM

Cardiac hypertrophy is defined as an increase in heart mass through growth of individual cardiomyocytes rather than an increment in cardiomyocyte. Physiological and pathological hypertrophy are two types of hypertrophy. Cardiac hypertrophy as a risk factor for heart failure, is a compensatory response that occurs as a result of hemodynamic overload (100–102). The process of hypertrophic cardiac remodeling is the response to the pathological insults, and ultimately cause impaired cardiac function (103, 104). Diabetic patients with impaired cardiac function are prone to the development of DCM. According to the Strong Heart study and the Cardiovascular Health study, they found that cardiac hypertrophy was often accompanied by cardiac systolic and/or diastolic function, suggesting a link between DM and cardiac hypertrophy (103, 105). In addition, diabetes-induced cardiac hypertrophy has been obtained from animal studies, which showed the increased ratio of heart weight to body weight (HW/BW) and cardiomyocyte size and upregulated hypertrophic gene expression (106).

Evidence for the role of HDACs in DCM is limited, but numerous studies revealed that HDACs contribute to the cardiac hypertrophy. Class I HDACs are generally identified pro-hypertrophic. Class I selective HDAC inhibitors have been reported to be efficacious agents, which could block cardiac hypertrophy induced by angiotensin II infusion and aortic banding (107). Similar results have been reported in other studies, that HDAC inhibitors (trichostatin A or scriptaid) ameliorated the cardiac hypertrophy induced by aortic banding (108). All this amount of evidence indirectly supported that HDACs contribute to the cardiac hypertrophy. Further studies disclosed that sodium butyrate which was a specific HADC inhibitor reduced heart/tibia ratio and areas of cardiomyocytes, suggesting that sodium butyrate lessened cardiac hypertrophy in the diabetic mice model. Adult mice were injected intraperitoneally with streptozotocin (STZ, 200mg/kg) to establish the diabetic mice model. In addition, sodium butyrate lessened cardiac hypertrophy which was associated with reducing interstitial fibrosis, relieving the apoptosis and stimulating angiogenesis in STZ-injected diabetic mice. These results have demonstrated that HDAC inhibitor reduced cardiac hypertrophy which in turn preventing diabetic mice from progressing to DCM (25). In contrast, class IIa HDACs have been identified as negative regulators of cardiac hypertrophy, by suppressing hypertrophic gene transcription (93, 109). Taken together, these evidences indirectly proved that HDACs dulled the occurrence of DCM by regulating cardiac hypertrophy.

**Figure 1:**
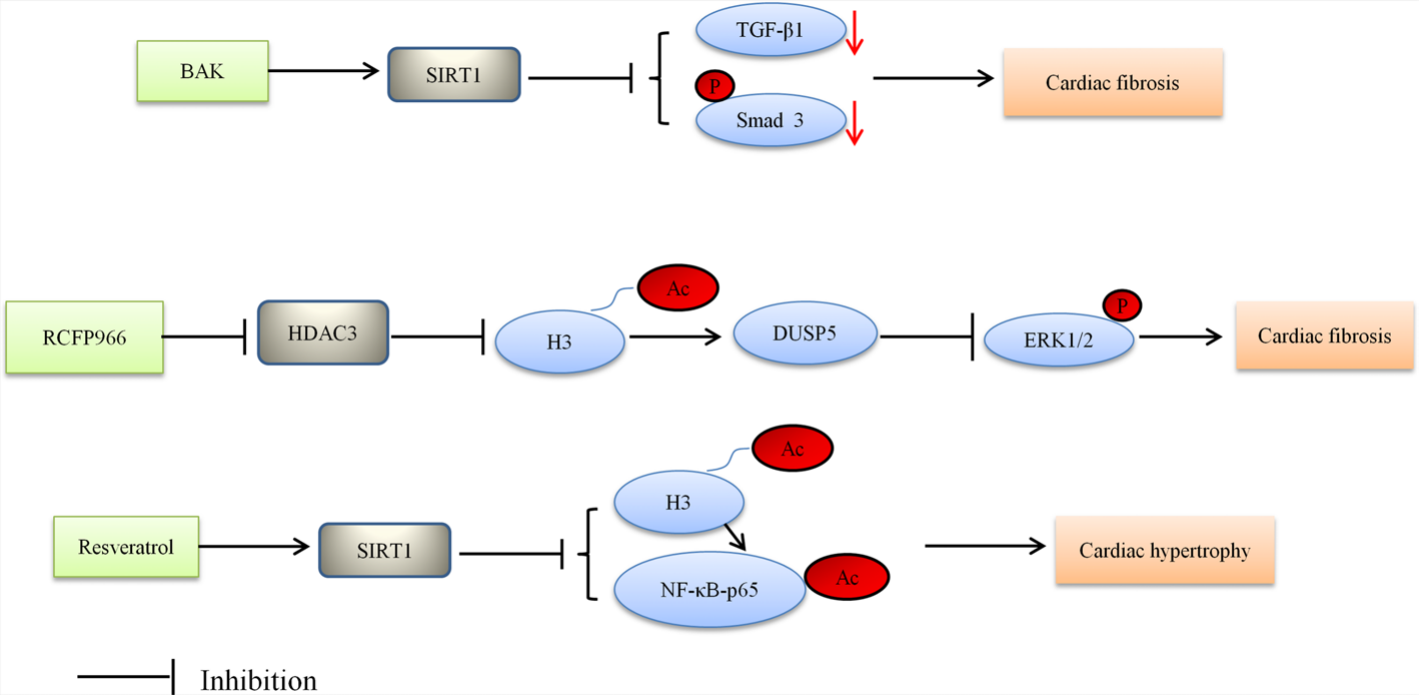
Proposed model depicting regulation and the role of HDACs in cardiac fibrosis and hypertrophy, and thus alleviating DCM. BAK, bakuchiol; Ac, acetylation; P, phosphorylation.

Based on Bagul’s study, SIRT1 activation by resveratrol led to deacetylation of both NF-κB-p65 and H3. SIRT1 activation decreased binding of NFκB-p65 to DNA, and lessened cardiac hypertrophy and oxidative stress, thereby ultimately blunting the development of DCM (110). The aforementioned study also suggested that oxidative stress may affect the physiology of the diabetic heart. There is some evidence showing the effect of oxidative stress on cardiac abnormalities including cardiac hypertrophy (110). These results suggest that HDACs are regulators of cardiac hypertrophy in the development of DCM. **Figure 1** depicted the role of HDACs regulated cardiac fibrosis and hypertrophy, and thus alleviating DCM. HDAC inhibitors reduced cardiac fibrosis and hypertrophy.

## HDACs, Oxidative Stress and DCM

Oxidative stress is widely considered to be one of the main contributors in the development and progression of diabetic cardiovascular complications, including DCM (36, 111). Under the conditions of DCM, the antioxidant factors such as superoxide dismutase (SOD) and glutathione peroxidase (GSH-Px) are sharply decreased in heart tissue, while the production of reactive oxygen species (ROS) is remarkably increased, which are responsible for cellular oxidative stress (112, 113). Excessive production of ROS can directly damage proteins, lipid membranes and DNA, oxidize lipids into harmful lipid peroxides, and increase the generation of reactive nitrogen species (RNS) (8, 36, 114, 115). Excessive generation of ROS can cause as well as activate several cellular stress-signaling and inflammatory pathways (116). Previous studies have reported that oxidative stress increased in human diabetic hearts (117).

HDACs have recently been reported to play a part in the pathological process of DCM, including oxidative stress. SIRT1 is the first member to be discovered in sirtuins and still the most studied one, especially as a potential target to treat cardiovascular diseases (56). SIRT1 mitigates oxidative stress and improves DCM *via* SIRT1/NF-κB-p65, SIRT1/FOXO1, SIRT1/NRF2 pathways. Resveratrol which was a polyphenolic compound used to be the potential prevention or therapy for DCM. Pankaj et al. found that SIRT1 activation by resveratrol led to deacetylation of both NF-κB-p65 at K310 and histone 3 at K9, thereby decreasing binding of NF-κB-p65 to DNA (110). The mice experiment showed that resveratrol ameliorated oxidative stress in diabetic mouse hearts depending on regulation of autophagic flux. Activation of SIRT1 led to deacetylation of FOXO1 and increased the transcriptional activity of FOXO1, ultimately enhanced the autophagy flux and protected diabetes-induced cardiac injury (118). Similarly, in Ren’s study, it was shown that curcumin treatment could alleviate DCM by modulating apoptosis and oxidative stress *via* the SIRT1-FOXO1 pathway (119). Given the oxidative stress seems a critical cause for the development of DCM, other anti-oxidative approaches have also been used to treat DCM, such as allisartan isoproxil, BAK and tetrahydrocurcumin (THC).

However, these anti-oxidative approaches respectively activated different SIRT1 signaling pathways (120–122). Previous researches have reported that BAK (a bioactive monoterpene phenol) and Allisartan isoproxil (a new nonpeptide angiotensin II receptor blocker [ARB] precursor drug) play crucial role in DCM by attenuating myocardial oxidative damage *via* activating the SIRT1/NRF 2 signaling pathway (91, 123). Furthermore, another research by Li et al. found that THC treatment could alleviate DCM by attenuating oxidative stress *via* activating the SIRT1 pathway (120). However, the SIRT1 pathway in Li’s study was not identical to those previously mentioned. SIRT1 activation by THC led to deacetylation of Ac-SOD2, while leading to produce SOD which is a vital molecule in maintaining ROS homeostasis. Thus, generation of ROS was reduced by THC *via* enhancing the SIRT1 pathway. These studies suggested that SIRT1 attenuates oxidative stress and improves DCM *via* SIRT1/NF-κB-p65, SIRT1/FOXO1, SIRT1/NRF2 pathways.

Among the seven different sirtuins, SIRT3 as a major protein deacetylase in mitochondria is involved in mediating cellular redox status, mitochondrial energetic and apoptosis (61). In Song’s study, it was demonstrated that SIRT3 deficiency increased ROS accumulation, aggravated hyperglycemia-induced mitochondrial damage, accelerated necroptosis, possibly activated the NLRP3 inflammasome, and ultimately exacerbated development of DCM in the mice (124). Elabela, another endogenous ligand of APJ, has been known as peptides. SIRT3 which is a downstream of APJ has been shown to protect DCM from oxidative stress-mediated cellular injury. Evidence from Li’s study indicated that the protective effects of Elabela in DCM are regulated by inhibition of oxidative stress *via* FOXO3a deacetylation (125).

**Figure 2:**
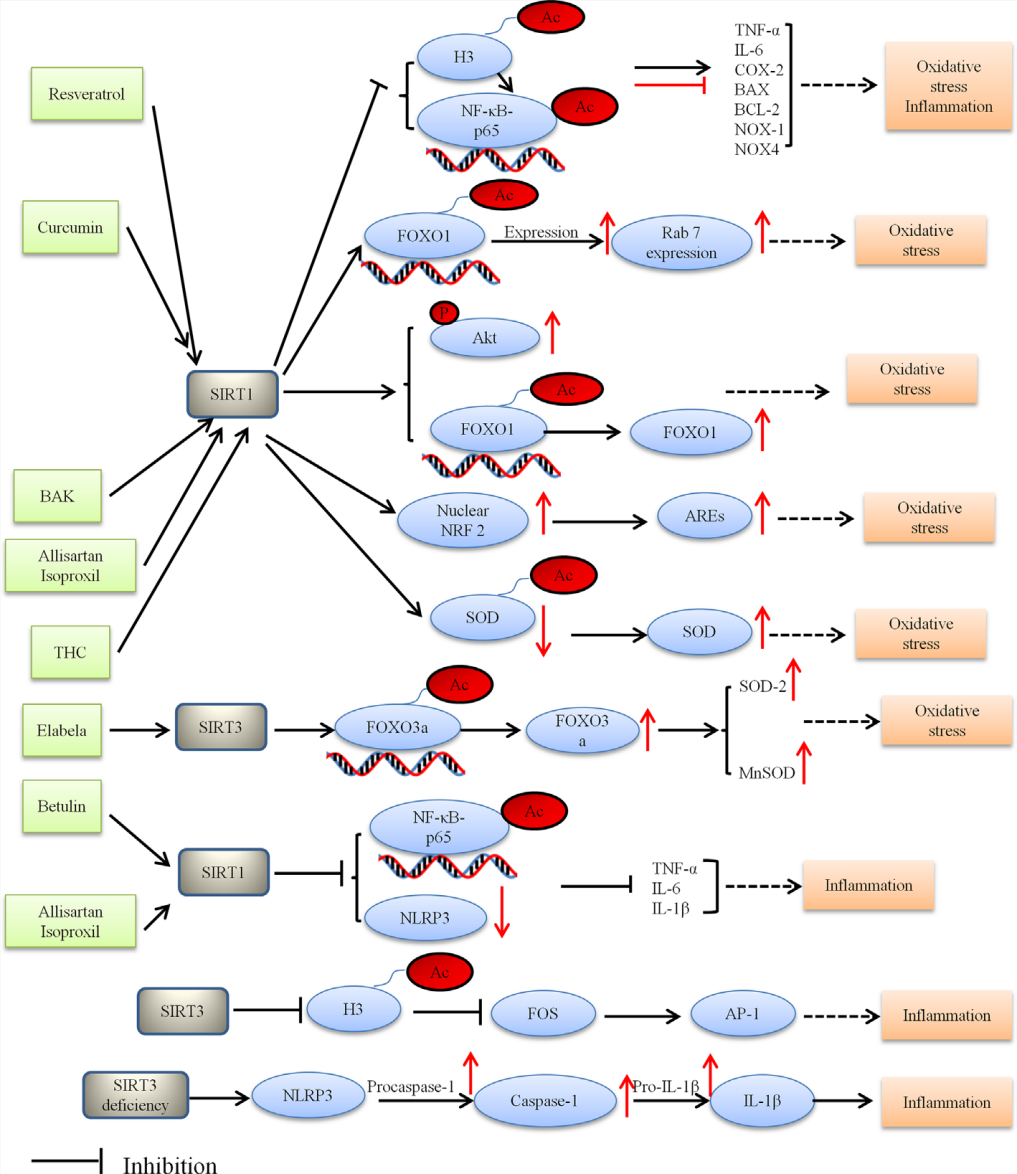
Proposed model depicting regulation and the role of HDACs in oxidative stress and inflammation, and thus alleviating DCM. BAK, bakuchiol; THC, tetrahydrocumin, AREs, antioxidant-responsive elements; Ac, acetylation; P, phosphorylation.

Taken together, HDACs potentially contribute to the pathogenesis of DCM, and they are also considered potential therapeutic target in DCM. **Figure 2** depicted the role of HDACs regulation oxidative stress and alleviating DCM.

## HDACs, Inflammation and DCM

Diabetic mellitus is a pro-inflammatory state (36, 126, 127), and many studies have revealed that cytokine of tissue concentrations increase in various diabetic mouse models, suggesting that inflammation plays an important role in the development of DCM. These studies demonstrated intramyocardial inflammation in DCM including increased inflammatory cells (infiltration of macrophages and leucocytes) and increased expression of inflammatory cytokines [leptin, TNF-α, TGF-β1, intercellular adhesion molecule 1 and vascular cell adhesion molecule 1 (ICAM-1 and VCAM-1), interleukin1 beta (IL-1β), interleukin 6 (IL-6), and interleukin 18 (IL-18)] (121, 122, 128–131).

Miao et al. revealed HDACs are associated with inflammation under diabetic conditions. High glucose can activate NF-κB and increase the expression of inflammation cytokines. *In vivo* experiment demonstrated that recruitment of NF-κB, histone acetyltransferases (HATs) and histone acetylation at the promoters of inflammatory genes was increased under diabetic conditions, which indicated that HDACs were associated with inflammation (132). The role of NF-κB in regulating inflammatory gene expression is well manifested (127, 132, 133). Likewise, NF-κB (a transcription factor) can be modified by HATs and HDACs leading to the upregulation of inflammatory genes (127). SIRT1 directly interacts with Rela/p65 and NF-κB subunits and inhibits NF-κB by deacetylating Rela/p65 at lysine 310, resulting in the nuclear translocation of NF-κB dependent on IκBa (134). Thereby, it decreased the expression of proinflammatory genes. Betulin and allisartan isoproxil alleviated DCM by attenuating inflammation *via* SIRT1/NF-κB pathway, however there are still differences between them (110, 123, 134, 135). NLRP3 inflammasome is a member of the NLRP superfamily. Inflammasomes are a group of protein complexes involved in inflammation, immunity and metabolic abnormalities of various diseases (135). In Wen’s study, it was demonstrated that Betulin (triterpene compound) plays anti-inflammation effect in the development of DCM by SIRT1 simultaneously activated NF-κB and NLRP3 (134). Moreover, Jin et al. reported that allisartan isoproxil alleviated DCM by attenuating diabetes-induced inflammation *via* the SIRT1/NF-κB pathway (123). From what has been discussed above, we draw the conclusion that SIRT1 reduced the inflammatory response and thus alleviated DCM. Taken together, SIRT1 is considered to be a potential intervention target in DCM (3, 136).

Several studies have uncovered multiple important links between the SIRT3 and inflammatory processes. SIRT3 knockout mice showed inflammation and cardiac fibrosis due to upregulation of AP-1 activity. SIRT3 inhibited FOS by deacetylating histone 3 at lysine K27. Palomer et al. found that SIRT3 regulated the proinflammatory and profibrotic responses of cardiac cells *via* the FOS/AP-1 pathway (137). Song et al. demonstrated that SIRT3 deficiency raised the expression of inflammation-related proteins containing NLRP3, caspase 1 p20, and interleukin-1β both *in vitro* and *in vivo*. Moreover, SIRT3 deficiency affected the development of DCM *via* the NLRP3 inflammasome (124). These results suggest that SIRT3 can be a molecular intervention target for the prevention and treatment of DCM. **Figure 2** depicted the role of HDACs regulation inflammatory and alleviating DCM.

## HDACIs and DCM

HDACs may be promising drug targets owing to their function in cell proliferation, cell cycle regulation, apoptosis, differentiation, metabolism, protein trafficking and DNA repair. HDAC inhibitors (HDACIs) are chemical compounds that block Zn^2+^-dependent HDAC enzymes involved in epigenetic modifications which regulate histone acetylation state. HDACIs have been approved by the US Food and Drug Administration (FDA) for clinical use, particularly for cancer treatment (138). Moreover, emerging studies indicated that epigenetic regulation of histone acetylation state may also own a potential for clinical application in the treatment of cardiovascular disease (139).

Currently, five HDACIs that are structurally divided into hydroxamic acid derivates (e.g., trichostatin A), short chain fatty (aliphatic) acids (e.g., sodium butyrate), cyclic peptides, benzamides and sirtuin inhibitors have been approved and are being used globally (140–142). While currently available HDACIs are largely non-selective (pan- HDACIs), the effects of HDACIs are usually studied by examining changes in bulk histone acetylation or the therapeutic effects observed in experimental model or in clinical trials (38). **Table 2** summarizes some progress of HDACIs related to treatment of cardiomyopathy regarding their anti-fibrotic and anti-hypertrophy effects. Therefore, more well-designed studies exploring the potential of selective HDACIs for DCM treatment is needed.

**Table 2:** Evidence showing the potential effect of inhibitors of HDACs in cardiomyopathy.

HDACI	HDAC Target	Cell or animal model	Effect	Reference
MPT0E014	Class I/IIb	Isoproterenol induced HF rats	Attenuated cardiac fibrosis	Kao et al. (99)
RGFP966	HDAC3	Type 1 diabetes OVE26 mice	Ameliorated cardiac fibrosis	Xu et al. (26)
Sodium butyrate	Nonspecific	Streptozotocin-induced diabetes mice	Ameliorated cardiac fibrosis and hypertrophy	Chen et al. (25)
SK-7041	Class I	Aortic banding (AB) mice	Prevented cardiac hypertrophy	Kee et al. (107)
Trichostatin A	Nonspecific	Aortic banding (AB) mice	Ameliorated cardiac hypertrophy	Kong et al. (108)
Scriptaid	Nonspecific	Aortic banding (AB) mice	Ameliorated cardiac hypertrophy	Kong et al. (108)

## Future Research Direction

The current evidence indicates that HDACs are involved in several biological pathways relevant to the pathogenesis of DCM as presented in **Figures 1**, **2**. However, more research is required to better understand the roles of HDACs in the pathogenesis of DCM and the mechanism that regulate them, and address the curative potential in treating DCM. For instance, some HDACs outside of the nucleus can also be post-translationally modified, which can subsequently alter the protein function. Exploring the proteins outside of the nucleus would therefore be a worthwhile endeavor to further understand the potential role of HDACs in DCM. HDACs do not solely remove acetyl residues from proteins but can also remove other acyl modifications, which provide better understanding of the mechanism. Likewise, assessing the relationship between HDACs and diabetes in heart failure may offer important insight into novel mechanisms for DCM. Nevertheless the currently available evidence provides a strong rationale for continuing preclinical studies and initiating clinical trials, with the ultimate purpose of testing the clinical utility of HDACs in DCM.

## Conclusion

Increasing evidence from *in vitro* and *in vivo* revealed that HDACs plays a critical role in the pathogenesis of DCM, suggesting that HDACs could be molecular intervention targets for the prevention and treatment of DCM. However, more endeavors are needed to further understand the roles of HDACs in the pathogenesis of DCM and the mechanism that regulate them. A comprehensive understanding of the mechanism of HDACs may provide a novel option for the prevention and treatment of DCM.

## Author Contributions

XK, XJ and GL prepared the initial draft of the manuscript. ZL, ZY, ML, BC and CJ edited the manuscript for intellectual content. Figures and Tables were created by XK and GL. All authors contributed to the article and approved the submitted version.

## Funding

This work was supported by the Science and Technology Program of Guangzhou (Grant sponsor: GL; Grant no.202002030252), the Science Foundation of Guangdong Second Provincial General Hospital (Grant sponsor: GL; Grant no.YY2018-002), and Doctoral workstation foundation of Guangdong Second Provincial general Hospital (Grant sponsor: XK; Grant no. 2021BSGZ008).

## Conflict of Interest

The authors declare that the research was conducted in the absence of any commercial or financial relationships that could be construed as a potential conflict of interest.
